# Dataset of the volatile compounds detected in unmarinated and marinated grilled ruminant meats with novel unfiltered beer-based marinades to improve their nutritional quality, safety, and sensory perception

**DOI:** 10.1016/j.dib.2019.104622

**Published:** 2019-10-07

**Authors:** Natalia P. Vidal, Charles Manful, Thu H. Pham, Evan Wheeler, Peter Stewart, Dwayne Keough, Raymond Thomas

**Affiliations:** a School of Science and the Environment, Boreal Ecosystem Research Initiative, Grenfell Campus, Memorial University of Newfoundland, Corner Brook, A2H 5G4, Canada; b Algoma University, 1520, Queen St E, Sault Ste. Marie, ON, P6A 2G4, Canada

**Keywords:** Headspace composition, SPME-GC/MS, Unfiltered beer-based marinated meat, Maillard reaction and lipid oxidation products, Volatile terpenes, Sulphur derivative compounds

## Abstract

The objective of this data in brief article is to present the associated data set regarding the published paper Novel unfiltered beer-based marinades to improve the nutritional quality, safety, and sensory perception of grilled ruminant meats in Food Chemistry [1]. Grilling is a popular cooking method; however, the high temperatures required can modify grilled meat quality and safety. In this data set, we include 5 tables containing the volatile composition of unmarinated and marinated grilled ruminant meat (beef and moose). Novel unfiltered beer-based marinades infused with herbs and spices were used for meat marination, and the volatiles present in the meat following grilling extracted by solid phase microextraction and subsequently analysed by gas chromatography/mass spectrometry (SPME-GC/MS). The volatile profile includes alcohols, aldehydes, ketones, acids, esters, alkylfurans, nitrogenated compounds, terpenes (mono-, sesqui- and oxygenated terpenes), sulfur derivatives, benzene derivatives, and phenol derivatives. This dataset provides valuable information on meat volatile composition useful to understand certain aspects of the quality and safety of grilled meat following preparation with unfiltered beer-based marinades. For more insight please see [1].

**Table:** Specifications Table

Subject	Agricultural and Biological Sciences
Specific subject area	Food Science
Type of data	Tables
How data were acquired	Data were acquired by the extraction of the volatile metabolites in the headspace of unmarinated and marinated grilled moose and beef meat by solid phase microextraction and the subsequent analysis by Gas Chromatography/Mass Spectrometry (SPME-GC/MS). Volatile compounds detected in the samples were semi-quantified based on the area counts x 10^−6^ of the base peak.
Data format	• Analysed • Raw
Parameters for data collection	Headspace compounds of the ground muscle of unmarinated and marinated grilled moose and beef meat was extracted by solid phase microextraction (SPME) with a Divinylbenzene/Carboxen/Polydimethylsyloxane (DVB/CAR/PDMS) coated fibre for 60 mins. GC-MS analysis of the volatiles was done using a Trace 1300 gas chromatography coupled to a TSQ 8000 Triple Quadrupole mass spectrometer.
Description of data collection	Volatile compounds in the samples were semi-quantified based on the area counts x 10^−6^ of the base peak. Compounds with lower abundances than 10,000 area counts were considered as traces. Three replicates (n = 3) were employed per experimental treatment. One-way analysis of variance (ANOVA) was used to determine if there were significant differences between the volatile compounds observed in marinated and unmarinated moose and beef samples. Where treatment effects were significant, the means were compared with Fisher's Least Significant Difference (LSD), α = 0.05.
Data source location	Institution: Memorial University of Newfoundland City/Town/Region: Corner Brook, NL Country: Canada
Data accessibility	Raw data are available within this article as supplementary material
Related research article	Author's name: Natalia P. Vidal, Charles Manful, Thu H. Pham, Evan Wheeler, Peter Stewart, Dwayne Keough, Raymond Thomas Title: Novel unfiltered beer-based marinades to improve the nutritional quality, safety, and sensory perception of grilled ruminant meats Journal: Food Chemistry DOI: https://doi.org/10.1016/j.foodchem.2019.125326

**Table:** 

**Value of the Data** • Data set contains the volatile composition of unmarinated and marinated grilled meats (moose and beef steaks) useful to determine the effects of unfiltered beer-based marinades on the quality, safety, and consumer sensory preferences of grilled ruminant meats. • This data could be used as a reference for the identification of volatile compounds coming from lipid oxidative processes, Maillard reaction, and beer-based marinades infused with herbs and spices. • The data will help to understand the usefulness of using unfiltered beer-based marinates to enhance the quality, safety and consumer sensory perception of grilled meat.

## 1 Data

The dataset contains more than 80 volatile compounds divided into five Tables. Table 1 shows the alcohols, aldehydes, alkylfurans and acids; Table 2: nitrogenated compounds; Table 3: terpenes; Table 4: sulphur derivatives and Table 5: combination of short chain acids, esters, ketones and alcohols detected in the headspace of unfiltered beer-based marinated grilled beef and moose meat by Solid Phase Micro-Extraction- Gas Chromatography/Mass Spectrometry (SPME-GC/MS). The statistical significance between marinated and unmarinated moose and beef samples is also presented. The raw data file is included as supplementary material in this article.

**Table 1:** Alcohols, aldehydes, alkylfurans and acids detected in the headspace of unfiltered beer-based marinated grilled beef and moose meat.

Compounds (MW)	Bp	UB	BM	BS	Sig.	UM	MM	MS	Sig.
*Alcohols*									
1-Pentanol (88)^†1^	42	6.9 ± 0.9	5.9 ± 0.2	6.1 ± 3.0	*ns*	8.9 ± 2.3	14.0 ± 2.8	12.8 ± 5.1	*ns*
1-Hexanol (102)^†1^	56	4.4 ± 0.5^b^	6.6 ± 0.3^a^	3.7 ± 0.3^b^	*	4.4 ± 0.7^b^	10.7 ± 1.8^a^	6.4 ± 0.1^b^	*
1-Heptanol (116)^†1^	56	3.8 ± 1.6	1.6 ± 1.5	2.7 ± 0.3	*ns*	2.5 ± 0.3^b^	4.2 ± 0.1^a^	1.7 ± 0.3^b^	**
1-Octen-3-ol (128)^†1^	57	20.4 ± 1.5^a^^b^	14.8 ± 0.5^b^	21.8 ± 2.1^a^	*	24.1 ± 2.1	18.0 ± 3.0	15.9 ± 2.3	*ns*
*Aldehydes*									
2-Methylpropanal (72)^2,3^	43	109.3 ± 7.2^a^	58.1 ± 1.5^b^	53.2 ± 2.0^b^	**	33.9 ± 12.5	*-*	*-*	**
3-Methylbutanal (86)^2,3^	41	17.3 ± 0.1^a^	7.6 ± 1.5^b^	10.3 ± 0.3^b^	*	15.9 ± 2.9	15.3 ± 6.7	14.6 ± 1.5	*ns*
2-Methylbutanal (86)^2,3^	57	39.5 ± 2.3^a^	11.5 ± 3.5^b^	7.2 ± 1.6^b^	*	31.0 ± 1.8^a^	13.5 ± 3.9^b^	5.1 ± 0.7^b^	*
Hexanal (100)^†1^	56	30.4 ± 0.6	17.7 ± 1.5	34.2 ± 5.8	*ns*	22.0 ± 5.7^a^	8.2 ± 0.7^b^	7.6 ± 0.6^b^	*
Heptanal (114)^†1^	44	12.3 ± 1.0^a^	4.3 ± 0.2^b^	4.9 ± 0.5^b^	**	14.4 ± 1.2^a^	4.2 ± 1.2^b^	3.3 ± 0.8^b^	**
Octanal (128)^†1^	43	10.8 ± 4.6	7.7 ± 0.6	12.2 ± 1.1	*ns*	60.2 ± 6.3^a^	7.1 ± 2.6^b^	8.1 ± 0.3^b^	**
Nonanal (142)^†1^	41	75.7 ± 6.3^a^	29.5 ± 1.2^a^	53.4 ± 8.1^ab^	*	256.1 ± 19.4^a^	57.0 ± 13.1^b^	55.7 ± 6.4^b^	**
Decanal (156)^†1^	41	4.7 ± 1.1	3.3 ± 1.2	5.4 ± 0.3	*ns*	10.5 ± 0.8	9.7 ± 2.6	10.3 ± 2.1	*ns*
Benzaldehyde (106)^1,3^	106	58.3 ± 15.2	18.6 ± 0.1	20.9 ± 2.3	*ns*	96.0 ± 6.0^a^	64.6 ± 12.1^b^	45.1 ± 4.7^b^	*
Benzeneacetaldehyde (120)^2^	91	53.0 ± 18.6	51.2 ± 16.5	9.9 ± 1.9	*ns*	20.9 ± 4.3	15.0 ± 2.6	15.4 ± 2.8	*ns*
2-Methyl-2-pentenal (98)^1^	41	*-*	*-*	*-*		2.7 ± 1.3^b^	10.6 ± 1.2^a^	8.8 ± 1.4^a^	*
(*E*)-2-octenal (126)^†1^	41	0.1 ± 0.1^c^	9.5 ± 1.1^a^	6.9 ± 0.3^b^	**	12.9 ± 1.5^c^	26.3 ± 5.6^b^	106.1 ± 3.5^a^	**
(*E*)-2-nonenal (140)^†1,3^	41	2.6 ± 1.0	3.0 ± 0.4	1.8 ± 0.2	*ns*	0.8 ± 0.1	1.2 ± 0.5	0.8 ± 0.5	*ns*
(*E*)-2-decenal (154)^1^	41	4.1 ± 2.8	6.1 ± 1.2	2.4 ± 0.1	*ns*	0.4 ± 0.3^b^	5.5 ± 1.2^a^	0.4 ± 0.2^b^	**
*Alkylfurans*									
2-Ethylfuran (96)^1^	81	0.8 ± 0.2^a^	0.4 ± 0.0^b^	0.4 ± 0.1^b^	*	2.2 ± 0.4^a^	0.7 ± 0.1^b^	0.3 ± 0.1^b^	**
5-Pentylfuran (138)^1^	81	9.7 ± 1.6	5.2 ± 0.9	5.1 ± 0.3	*ns*	22.3 ± 4.8^c^	7.7 ± 0.6^b^	7.2 ± 1.2^b^	*
5-Heptylfuran (167)^1^	81	1.3 ± 0.5	0.5 ± 0.1	0.7 ± 0.1	*ns*	1.3 ± 0.3	2.4 ± 1.6	0.1 ± 0.0	*ns*
*Acids*									
3-Methylbutanoic acid (102)^2,3^	60	2.1 ± 0.1	0.9 ± 0.2	4.0 ± 1.6	*ns*	1.3 ± 0.4^b^	4.6 ± 0.9^a^	0.7 ± 0.1^b^	**
Hexanoic acid (116)^†1,3^	60	49.9 ± 3.1^a^	38.9 ± 7.3^a^	14.8 ± 1.0^b^	*	44.5 ± 2.1^a^	29.7 ± 1.6^b^	24.0 ± 4.9^b^	*
Heptanoic acid (130)^†1^	60	50.1 ± 2.7^a^	15.3 ± 2.0^b^	31.2 ± 0.2^ab^	*	48.49 ± 2.8^a^	29.5 ± 1.8^b^	21.7 ± 3.9^b^	**
Octanoic acid (144)^†1,3^	60	37.4 ± 12.7	28.2 ± 5.9	12.7 ± 2.6	*ns*	12.92 ± 1.0^b^	31.0 ± 2.3^a^	27.6 ± 2.0^a^	**
Nonanoic acid (158)^†1^	60	47.5 ± 6.1^a^	43.6 ± 0.8^a^	6.9 ± 3.7^b^	**	8.19 ± 0.1^b^	44.4 ± 2.2^a^	11.2 ± 2.1^b^	**

Values (means ± standard errors; n = 3) represent the abundances, expressed as area counts of their mass spectra base peak (Bp) divided by 10^6^, together with the molecular weight (MW). Sig: statistical significance of the samples. *ns*: no significant difference; *: significant difference (p < 0.05); **: significant difference (p < 0.01). Rows with different letters show significant differences between treatments at LSD = 0.05. ^†^: positively identified by comparison with standards mass spectrum; ^1^: compounds coming mainly from lipid oxidation; ^2^: compounds coming from the Strecker degradation; ^3^: compounds coming from the beers (Liu, 2015). [UB, UM] = unmarinated grilled beef and moose; [BM, MM] = Indian Session Ale unfiltered beer-based marinated grilled beef and moose; [BS, MS] = wheat Ale unfiltered beer-based marinated grilled beef and moose.

**Table 2:** Nitrogenated compounds detected in the headspace of unfiltered beer-based marinated grilled beef and moose meat.

Nitrogenated compounds (MW)	Bp	UB	BM	BS	Sig.	UM	MM	MS	Sig.
Pyrrole (67)	67	10.8 ± 3.2^a^	2.4 ± 0.2^ab^	1.9 ± 0.4^b^	*	4.4 ± 0.9^ab^	5.6 ± 1.2^a^	1.9 ± 0.2^b^	*ns*
Pyridine (79)^†^	79	14.8 ± 2.0^a^	*-*	0.1 ± 0.0^b^	*	6.2 ± 1.6^b^	13.0 ± 1.2^a^	5.2 ± 1.3^b^	*
3-Methylpyridine (93) or isomer	93	4.8 ± 2.5	1.1 ± 1.0	*-*	*ns*	3.8 ± 1.0	17.3 ± 8.9	4.5 ± 0.8	*ns*
2-Methylpyrimidine (94) or isomer	94	39.4 ± 4.4^a^	7.9 ± 2.5^b^	8.2 ± 1.5^b^	*	22.5 ± 7.7	14.6 ± 2.6	7.7 ± 1.6	*ns*
2,6-Dimethylpyrazine (108) or isomer	108	139.9 ± 5.8^a^	18.8 ± 10.1^b^	24.6 ± 18.6^b^	*	109.4 ± 19.1^a^	41.0 ± 8.8^b^	19.3 ± 1.6^b^	**
2,3-Dimetylpyrazine (108)	108	20.1 ± 5.5	3.8 ± 1.9	4.8 ± 2.9	*ns*	9.7 ± 1.3^a^	4.5 ± 1.0^b^	2.0 ± 0.5^b^	**
2-Ethyl-6-methylpyrazine (122) or isomer	121	6.0 ± 1.6^b^	20.8 ± 1.5^a^	14.4 ± 1.4^a^	**	3.6 ± 1.0^b^	22.8 ± 5.5^a^	0.5 ± 0.1^b^	**
2-Ethyl-5-methylpyrazine (122) or isomer	121	44.0 ± 1.7^a^	11.8 ± 6.7^b^	3.6 ± 1.2^b^	*	31.1 ± 7.1^a^	0.5 ± 0.2^b^	5.1 ± 2.5^b^	**
Ethyl-methylpyrazine (122) isomer	122	127.8 ± 8.1^a^	9.3 ± 2.3^b^	15.9 ± 8.3^b^	*	3.6 ± 1.0^b^	22.8 ± 5.5^a^	0.4 ± 0.2^b^	**
1-(1-Pyrrol-2-yl)-ethanone (109)	94	8.5 ± 2.6	20.7 ± 6.2	10.4 ± 2.2	*ns*	*-*	12.3 ± 1.5^a^	*-*	**
2-Ethyl-3,5-dimethylpyrazine (136) or isomer	135	65.7 ± 11.4^a^	6.8 ± 2.3^b^	10.8 ± 6.0^b^	*	61.7 ± 9.3^a^	44.3 ± 5.7^b^	27.5 ± 1.4^b^	**
3-Ethyl-2,5-dimethylpyrazine (136) or isomer	135	7.9 ± 0.5^a^	0.9 ± 0.3^b^	1.7 ± 1.0^b^	*	7.8 ± 2.7	4.9 ± 1.3	3.7 ± 1.3	*ns*
Ethyl-dimethylpyrazine (136) isomer	135	7.9 ± 0.5^a^	2.6 ± 1.7^ab^	0.7 ± 0.2^b^	*	6.5 ± 1.2	4.0 ± 1.4	3.6 ± 1.1	*ns*
Maltol (126)	126	1.1 ± 0.8^b^	10.2 ± 2.1^a^	1.7 ± 1.6^b^	*	0.2 ± 0.1	3.8 ± 1.0	3.2 ± 2.9	*ns*
Indole (117)	117	5.6 ± 1.0	2.8 ± 1.0	1.3 ± 1.0	*ns*	2.9 ± 1.1	5.0 ± 0.2	3.4 ± 1.1	*ns*
3,5-Diethyl-2-methylpyrazine (150) or isomer	149	3.3 ± 1.0	1.1 ± 0.6	0.3 ± 0.2	*ns*	3.1 ± 1.0	4.0 ± 1.2	3.3 ± 1.3	*ns*
2,5-Dimethyl-3-(3-methylbutyl)-pyrazine (178)	122	7.6 ± 1.5^a^	1.7 ± 0.3^ab^	1.0 ± 0.5^b^	*	4.6 ± 0.4	4.8 ± 1.1	4.2 ± 1.2	*ns*

Values (means ± standard errors; n = 3) represent the abundances, expressed as area counts of their mass spectra base peak (Bp) divided by 10^6^, together with the molecular weight (MW). Sig: statistical significance of the samples. *ns*: no significant difference; *: significant difference (p < 0.05); **: significant difference (p < 0.01). Rows with different letters show significant differences between treatments at LSD = 0.05. ^†^: positively identified by comparison with standards mass spectrum; [UB, UM] = unmarinated grilled beef and moose. [BM, MM] = Indian Session Ale unfiltered beer-based marinated grilled beef and moose; [BS, MS] = wheat Ale unfiltered beer-based marinated grilled beef and moose.

**Table 3:** Terpenes compounds detected in the head space of unfiltered beer-based marinated grilled beef and moose meat.

Terpenes (MW)	Bp	UB	BM	BS	Sig.	UM	MM	MS	Sig.
*Monoterpenes hydrocarbons*									
Pinene (136) isomer	93	*-*	1.4 ± 0.0^a^	1.7 ± 0.2^a^	**	0.4 ± 0.1^b^	6.9 ± 1.2^a^	7.2 ± 2.0^a^	*
α-Pinene (136)	93	*-*	5.8 ± 1.2^a^	7.1 ± 0.5^a^	**	0.9 ± 0.4^c^	52.7 ± 6.2^b^	159.4 ± 17.4^a^	**
Camphene (136)	93	*-*	*-*	*-*		0.3 ± 0.2^b^	3.3 ± 0.4^b^	8.6 ± 2.0^a^	**
o-Cymene (134)	119	*-*	1.4 ± 0.2^a^	1.4 ± 0.1^a^	**	0.3 ± 0.1^b^	5.0 ± 0.2^ab^	8.9 ± 2.8^a^	*
3-Carene (136) isomer	93	*-*	9.7 ± 2.0a	12.1 ± 0.8^a^	**	4.3 ± 1.4^c^	69.7 ± 9.5^b^	238.4 ± 38.7^a^	**
a-Myrcene (136)	93	*-*	14.3 ± 2.8a	16.3 ± 1.4^a^	**	2.8 ± 0.9^c^	71.6 ± 6.8^b^	165.4 ± 35.3^a^	**
Terpinene (136) isomer	93	1.0 ± 0.4	76.5 ± 17.1a	84.9 ± 2.5^a^	**	5.0 ± 2.5^b^	380.3 ± 23.8^ab^	1021.2 ± 387.4^a^	*
p-Cymene (134) or isomer	119	*-*	*-*	*-*		*-*	11.4 ± 1.0	21.2 ± 8.3	*ns*
Cymene (134) isomer	119	0.3 ± 0.2	30.0 ± 3.9b	41.9 ± 3.8^a^	**	6.7 ± 1.6^b^	190.7 ± 9.9^a^	186.1 ± 54.0^a^	**
Limonene (136)	68	4.3 ± 0.9	69.2 ± 11.3a	72.4 ± 4.9^a^	**	15.8 ± 5.3^b^	309.7 ± 27.1^ab^	629.3 ± 189.1^a^	*
Terpinene (136) isomer	93	*-*	*-*	*-*		31.1 ± 7.1^a^	0.5 ± 0.2^b^	7.6 ± 0.5^a^	**
Elemene (204) isomer	121	*-*	*-*	*-*		*-*	37.4 ± 1.2^a^	184.9 ± 43.2^b^	*
*Oxygenated monoterpenes*									
Linalool (154)	71	2.4 ± 0.7	14.6 ± 0.7	17.5 ± 0.7	**	2.1 ± 0.2^b^	58.6 ± 9.0^a^	72.6 ± 16.6^a^	**
Endo-borneol (154)	95	*-*	3.4 ± 0.1^b^	5.7 ± 0.9^a^	**	0.8 ± 0.1	1.2 ± 0.5	0.8 ± 0.5	*ns*
Terpinen-4-ol (154)	71	1.8 ± 1.4	4.5 ± 0.2^ab^	8.1 ± 0.9^a^	*	2.4 ± 1.8^b^	24.0 ± 5.3^a^	31.6 ± 6.0^a^	*
Terpineol (154) isomer	59	0.8 ± 0.4	3.1 ± 0.0^ab^	5.7 ± 1.1^a^	*	0.8 ± 0.7^b^	13.0 ± 2.9^a^	20.7 ± 3.1^a^	**
Carvacrol (150) isomer	135	0.3 ± 0.1	3.8 ± 0.7^b^	7.9 ± 1.2^a^	**	*-*	28.5 ± 5.0^a^	27.9 ± 9.8^a^	**
Carvacrol (150)	135	*-*	196.2 ± 47.0^a^	188.6 ± 28.2^b^	**	*-*	583.7 ± 141.0^a^	686.6 ± 229.9^a^	*
*Sesquiterpenes*									
Coapene (204) isomer	105	*-*	*-*	0.4 ± 0.0^a^	**	*-*	2.7 ± 0.2	7.0 ± 2.9	*ns*
α-Copaene (204)	105	*-*	4.0 ± 0.0^b^	7.4 ± 0.1^a^	**	0.3 ± 0.0^c^	50.0 ± 2.2^a^	27.4 ± 10.7^b^	**
Cariophyllene (204) isomer	91	*-*	1.2 ± 0.0^b^	2.5 ± 0.2^a^	**	*-*	44.2 ± 35.0	23.6 ± 8.9	*ns*
Cariophyllene (204)	92	*-*	39.4 ± 1.4^b^	74.1 ± 4.9^a^	**	0.3 ± 0.1^c^	519.4 ± 41.7^b^	1812.7 ± 152.4^a^	**
α-Guaiene (204)	91	0.3 ± 0.1	0.9 ± 0.1^b^	2.0 ± 0.3^a^	**	0.8 ± 0.0	7.5 ± 1.6	15.6 ± 6.1	*ns*
Humulene (204)	93	0.4 ± 0.0	2.2 ± 0.2^b^	4.0 ± 0.2^a^	**	*-*	22.9 ± 1.9^b^	91.4 ± 10.0^a^	**
Muurolene (204) isomer	105	*-*	*-*	*-*		*-*	2.1 ± 0.1^ab^	6.1 ± 2.1^a^	**
α-Bisabolene (204)	41	*-*	0.9 ± 0.0	3.8 ± 2.0	*ns*	*-*	11.1 ± 0.4^b^	30.3 ± 7.0^a^	**

Values (means ± standard errors; n = 3) represent the abundances, expressed as area counts of their mass spectra base peak (Bp) divided by 10^6^, together with the molecular weight (MW). Sig: statistical significance of the samples. *ns*: no significant difference; *: significant difference (p < 0.05); **: significant difference (p < 0.01). Rows with different letters show significant differences between treatments at LSD = 0.05. [UB, UM] = unmarinated grilled beef and moose; [BM, MM] = Indian Session Ale unfiltered beer-based marinated grilled beef and moose; [BS, MS] = wheat Ale unfiltered beer-based marinated grilled beef and moose.

**Table 4:** Sulfur derivatives detected in the head space of unfiltered beer-based marinated grilled beef and moose meat.

Sulfur derivatives (MW)	Bp	UB	BM	BS	Sig.	UM	MM	MS	Sig.
Methanethiol (48)^1,2^	47	9.0 ± 3.5^b^	298.2 ± 25.8^a^	337.5 ± 11.7^a^	**	13.8 ± 7.4^c^	477.9 ± 28.4^a^	360.6 ± 6.6^b^	**
Dimethyl disulfide (94)^1^	94	3.7 ± 1.3^a^	0.4 ± 0.1^b^	0.3 ± 0.0^b^	*	2.9 ± 0.5^a^	0.6 ± 0.2^b^	0.2 ± 0.0^b^	**
Diallyl sulfide (114)^2^	45	*-*	*-*	*-*		0.7 ± 0.1^b^	7.0 ± 1.1^a^	4.6 ± 1.7^ab^	*
Allyl isothiocyanate (99)^2^	99	*-*	85.3 ± 11.7^a^	10.4 ± 3.9^b^	**	*-*	20.5 ± 3.3^a^	13.6 ± 2.1^a^	**
Dimethyl trisulfide (94)^1^	126	4.3 ± 3.0	*-*	*-*	*ns*	2.0 ± 1.4	*-*	*-*	*ns*
Diallyl disulfide (146)^2^	41	3.2 ± 0.7^b^	26.3 ± 0.7^a^	26.4 ± 3.8^a^	**	4.9 ± 0.3^b^	101.6 ± 6.8^a^	93.6 ± 7.6^a^	**
Dipropyl disulfide (150)^2^	150	*-*	1.2 ± 0.2^a^	1.1 ± 0.1^a^	**	*-*	6.4 ± 1.3^a^	7.2 ± 2.0^a^	*
2-Ethyl-1,3-dithiane (148)^2^	119	1.7 ± 0.3	1.8 ± 0.1	1.7 ± 0.1	*ns*	*-*	5.0 ± 0.7^b^	17.5 ± 2.3^a^	**
1-Allyl-2-isopropyldisulfane (148)^2^	41	1.6 ± 0.1^b^	7.4 ± 1.1^a^	6.2 ± 1.0^a^	**	2.9 ± 0.5^c^	31.2 ± 6.5^b^	47.8 ± 5.4^a^	**
Allyl trisulfide (178)^2^	73	*-*	6.3 ± 1.5^a^	6.7 ± 1.2^a^	**	*-*	6.9 ± 2.1^a^	13.3 ± 2.4^a^	**

Values (means ± standard errors; n = 3) represent the abundances, expressed as area counts of their mass spectra base peak (Bp) divided by 10^6^, together with the molecular weight (MW). Sig: statistical significance of the samples. *ns*: no significant difference; *: significant difference (p < 0.05); **: significant difference (p < 0.01). Rows with different letters show significant differences between treatments at LSD = 0.05. ^1^: compounds coming from the amino acids degradation; ^2^: compounds coming mainly from the unfiltered beer-based marinades. [UB, UM] = unmarinated grilled beef and moose; [BM, MM] = Indian Session Ale unfiltered beer-based marinated grilled beef and moose; [BS, MS] = wheat Ale unfiltered beer-based marinated grilled beef and moose.

**Table 5:** Combination of short chain acids, esters, ketones and alcohols detected in the headspace of unfiltered beer-based marinated grilled beef and moose meat.

Compounds (MW)	Bp	UB	BM	BS	Sig.	UM	MM	MS	Sig.
Acetic acid (60)	43	74.0 ± 60.3	60.7 ± 40.8	1.8 ± 0.0	*ns*	22.0 ± 1.7^b^	38.0 ± 3.4^a^	351 ± 6.1^ab^	*
Ethyl acetate (88)	43	0.1 ± 0.1^b^	71.7 ± 17.1^a^	77.0 ± 1.9^a^	**	22.0 ± 1.7^c^	134.5 ± 11.0^a^	104.5 ± 2.3^b^	**
Acetic anhydride (102)	43	107.8 ± 7.7^a^	53.4 ± 1.8^b^	58.0 ± 1.3^b^	**	39.1 ± 1.2^b^	251.2 ± 16.6^a^	61.9 ± 12.3^b^	**
2,3-Butanedione (86)	43	20.0 ± 2.7	15.6 ± 1.3	18.3 ± 1.3	*ns*	9.7 ± 2.3^b^	54.7 ± 4.5^a^	9.6 ± 4.4^b^	**
Ethanol (46)	45	0.7 ± 0.0^b^	2663.6 ± 96.8^a^	2760.8 ± 112.6^a^	**	0.0 ± 0.0^b^	3058.2 ± 130.3^a^	2800.2 ± 196.9^a^	**
1-Butanol, 3-methyl (88)	55	6.8 ± 5.5^c^	188.7 ± 54.4^b^	340.5 ± 30.5^a^	**	9.5 ± 6.0^c^	275.1 ± 31.0^b^	530.7 ± 78.1^a^	**
1-Butanol, 2-methyl (88)	57	2.4 ± 1.3^b^	177.7 ± 58.5^a^	311.9 ± 22.4^a^	*	4.5 ± 0.4^c^	64.3 ± 3.8^b^	162.5 ± 25.6^a^	**
Phenylethyl alcohol (122)	91	0.5 ± 0.3^c^	258.5 ± 108.2^b^	762.5 ± 107.5^a^	**	0.1 ± 0.0^c^	268.7 ± 15.9^b^	1851.4 ± 184.9^a^	**
3,5-Di-*tert-*butyl-4-hydroxybenzaldehyde (BHT-CHO) (234)	219	9.5 ± 4.2	20.4 ± 16.8	45.3 ± 4.7	*ns*	30.0 ± 3.0^a^	15.3 ± 2.1^b^	10.5 ± 0.2^b^	**

Values (means ± standard errors; n = 3) represent the abundances, expressed as area counts of their mass spectra base peak (Bp) divided by 10^6^, together with the molecular weight (MW). [UB, UM] = unmarinated grilled beef and moose; [BM, MM] = Indian Session Ale unfiltered beer-based marinated grilled beef and moose; [BS, MS] = wheat Ale unfiltered beer-based marinated grilled beef and moose. Sig: statistical significance of the samples. *ns*: no significant difference; *: significant difference (p < 0.05); **: significant difference (p < 0.01). Rows with different letters show significant differences between treatments at LSD = 0.05.

## 2 Experimental design, materials, and methods

### 2.1 Unfiltered beer-based marinade preparation and moose or beef meat marination

Two types of unfiltered beers (Indian session Ale and Wheat Ale) purchased from a local liquor store were selected for the preparation of the marinades. India session Ale (M) contained water, malted barley, and hops whereas Wheat ale beer (S) contained water, malted wheat, barley, orange, lemon, lime peel, coriander, cascade and Willamette hops. To 341 ml of each type of unfiltered beer, a mix of 1 g oregano, 1 g of parsley, 4 g of mustard, 21 g of salt, 8 g of pepper, 1 g of garlic, 25 ml of olive oil, 15 ml of vinegar and 25 g of fresh onions purchased from a local market were added to a food processor and the content homogenized and mixed thoroughly to obtain the beer-based marinade that was further employed to marinade the moose and beef meat samples. Beef and moose meat was obtained from a local market and from Newfoundland and Labrador Department of Natural Resources respectively. Moose and beef steaks were taken from 4 different animals to mitigate any inherent variability of the meat resource. Ethics approval for this study was granted by Memorial University Animal Care Committee as mandated by the Canadian Council on Animal Care and all the experiments were performed in accordance with relevant guidelines and regulations. Steaks (1 lb) of beef (B) and moose (M) meat from different batches were cut and divided into four replicates (n = 4) per treatment (n = 3). Each replicate was made from an independent batch of beer and ingredients. The steaks were divided into three groups as follow: control group (unmarinated, U), treatment group marinated with Indian Session Ale marinade (M) and treatment group marinated with Wheat Ale beer-based marinade (S). Meat marination was performed by adding 600 mL of each beer-based marinades to the beef and moose steaks for 12 hrs at 4 °C in zip lock closed plastic bags. The unmarinated samples (U, control) were kept under the same conditions as marinated ones until grilling time [1].

### 2.2 Cooking conditions

Beef and moose unmarinated (UB, UM) and marinated (BM,BS; MM,MS) samples were grilled at 200–250 °C for 25 minutes on a grill (Cuisinart® Gourmet 600B) reaching an internal temperature of 75 °C. In both types of meat, the unmarinated meat was cooked before the marinated ones. The barbeque was thoroughly cleaned between samples to avoid any possible contamination of marinade flavours. Meat samples were turned regularly during grilling. After grilling, each replicate was divided into two subsets. One subset was cut into two-inch cubes and used for sensory analysis, while the other subset was labeled and stored at −80 °C for chemical analysis [1].

### 2.3 Extraction of the volatile components by solid phase microextraction coupled to gas chromatography/mass spectrometry (SPME-GC/MS)

One gram of ground muscle of each sample was weighed and placed in 10 mL headspace glass vials. After 5 min of sample equilibration at 50 °C, a Divinylbenzene/Carboxen/Polydimethylsyloxane (DVB/CAR/PDMS) coated fibre with the following dimensions: 1 cm long, 50/30 μm film thickness (Supelco, Sigma-Aldrich, St. Louis, MO, USA), was inserted into the headspace of the sample vial and held there for 60 mins [2,3]. GC-MS analysis of the unmarinated and marinated beef and moose volatile composition was done using a Trace 1300 gas chromatography coupled to a TSQ 8000 Triple Quadrupole mass spectrometer (ThermoScientific, Brampton, ON, Canada). The extracted volatile compounds were separated using a ZB-5MS non-polar stationary phase column (30  m × 0.25 mm I.D., 0.25 μm film thickness) (Phenomenex, CA, USA) with He used as the carrier at a flow rate of 1 mL/min. After the extraction period, the fibre was desorbed for 10 min in the injection port. The operation conditions of the instrument were as follows: splitless mode was used for injection with a purge time of 5 min. The oven temperature was initially set at 50 °C (5 min hold) and then increased to 290 °C at 4 °C/min (2 min hold). Ion source and quadrupole mass analyzer temperatures were set at 230 and 150 °C respectively. The injector and detector temperatures were held at 250 and 290 °C respectively. Mass spectra were recorded at an ionisation energy of 70 eV, with data acquisition done in scan mode. After each sample desorption, the fibre was cleaned for 10 min at 250 °C in the conditioning station. Volatile compounds were identified by matching (matching factor > 80% used) the obtained mass spectra with those of available standards, and mass spectra obtained from commercial libraries NIST/EPA/NIH (version 2.2, ThermoScientific) or the scientific literature [2,3]. Volatile compounds in the samples were semi-quantified based on the area counts x 10^−6^ of the base peak. Compounds with lower abundances than 10,000 area counts were considered as traces. Although the chromatographic response factor of each compound is different, the area counts determined are useful for comparison of the relative abundance of each compound in the different samples analysed. Three replicates (n = 3) were employed per experimental treatment [1].

### 2.4 Statistical analysis

One-way analysis of variance (ANOVA) was used to determine if there were significant differences between the abundances of volatile compounds observed in marinated and unmarinated moose and beef samples. Where treatment effects were significant, the means were compared with Fisher's Least Significant Difference (LSD), α = 0.05, [1].
